# Are you alone? Measuring solitude in childhood, adolescence, and emerging adulthood

**DOI:** 10.3389/fpsyt.2023.1179677

**Published:** 2023-04-20

**Authors:** Alicia McVarnock, Tiffany Cheng, Laura Polakova, Robert J. Coplan

**Affiliations:** Department of Psychology, Carleton University, Ottawa, ON, Canada

**Keywords:** solitude, time alone, childhood, adolescence, emerging adulthood, measuring solitude

## Abstract

The goal of this review was to provide an overview of how solitude has been operationally defined and measured since the year 2000 in psychological studies of children, adolescents, and emerging adults. After applying exclusionary criteria, our review of the extant literature identified *n* = 19 empirical studies, which we grouped into three broad methodological categories: (1) experiments/manipulations (*n* = 5); (2) retrospective reports (*n* = 7); and (3) experience sampling measures (experience sampling methodology; *n* = 7). A review of these studies indicated considerable variation in how solitude is operationalized and measured. There is also a notable lack of studies measuring solitude in childhood. Implications for ‘what matters’ when assessing solitude are discussed, and we provide a series of suggestions for helping this research area move forward.

## Introduction

The study of solitude has a rich history in developmental psychology, with theoretical perspectives highlighting the potential costs and benefits of spending time alone. For example, excessive solitude has long been characterized as a cause of distress (1) and indicator of psychopathology (2). It was also commonly argued that because social connections are essential for healthy development and well-being, children spending frequent time alone are at increased risk of ‘missing out’ on benefits afforded by social interactions and relationships (3). Other perspectives have focused on the constructive role of solitary play for child development (4), solitary experiences as reprieve from social stresses (5), and the emergence of solitude as a domain for positive development in adolescence (6).

Many studies have explored the psychological aspects of solitude among children and youth over the last two decades, with a particular focus on the causes and consequences of time alone (22). The COVID-19 pandemic led to government-imposed containment strategies (e.g., lockdowns, social distancing), resulting in an overall increase in time spent alone (7). Such experiences have shone a brighter spotlight on the potential impacts of solitude on mental health and well-being in children, adolescents, and emerging adults (8). However, variations in how solitude is conceptualized, operationally defined, and measured have made it difficult to compare results across studies. Moreover, despite increased focus on aspects pertaining to the broad phenomenon of solitude (e.g., social withdrawal, peer exclusion, ostracism, loneliness, and aloneliness), few studies have assessed solitude itself. Accordingly, the goal of this review article was to provide an overview of how solitude has been operationally defined and measured since the year 2000 in psychological studies of children, adolescents, and emerging adults.

### Conceptualizations of solitude

There has been considerable variation in the psychological conceptualizations of solitude. For example, a common (and seemingly objective) perspective considers solitude as a *physical* separation from others. In this regard, Goffman (9) described solitude using the metaphor of being ‘off stage’ and removed from perceived social expectations and demands. However, as has been previously noted, there is no consensus among researchers as to the required minimum physical distance from others for an individual to be considered alone (10). Moreover, even within the criteria of being physically separated from others, further conceptual distinctions can still be made. In some cases, solitude is defined as necessitating a lack of accompanying activity, sometimes referred to as *pure* solitude (11) or being alone with one’s thoughts (12). In others, the central focus has been on characterizing and distinguishing among different activities that adolescents and young adults engage in alone [e.g., homework vs. watching videos, daydreaming vs. ruminating; (13)].

Other conceptualizations of solitude do not stipulate physical separation from others. From these perspectives, solitude occurs when we *feel* alone (14) and relates to our *perceived* social separation (15). Importantly, this allows for solitude to be experienced in the presence of others (i.e., alone in a crowd), such as sitting alone on a commuter train (16) or visiting an art gallery without a companion (17). To make matters more complicated, physical separation no longer implies a lack of social interaction. Advances in contemporary technology have made it commonplace to engage in computer-mediated interactions (including FaceTime) while physically alone (18). Indeed, Hipson et al. (13) recently reported that screentime (e.g., social media, texting, watching videos, playing video games) was the most common solitary activity among adolescents. In this regard, it has been recently suggested that solitude be reconceptualized as *non*-*communication* [i.e., not physically or virtually interacting with others; (19)]. Notably, adolescents have a nuanced conceptualization of the intersection between solitude and technology, defining different ‘degrees’ of solitude as a function of engagement in passive versus text-based versus audio-visual technologies (20).

Finally, there has been extensive research into the putative ‘causes’ of solitude in childhood and adolescence. For example, Rubin and Mills (21) distinguished between the processes of *active isolation* (children are forced into unwanted solitude due to peer rejection/exclusion) and *social withdrawal* (children remove themselves from opportunities for peer interaction). Asendorpf (22) later described different subtypes of social withdrawal, characterized by specific combinations of social approach and avoidance motivations. For example, s*hyness* (high approach; high avoidance) is characterized by an internal conflict between the desire to engage with others and socio-evaluative fears. Of note, shyness shares conceptual overlap (but is distinct from) anxiety (particularly social anxiety), which can also fuel solitary behavior (23). Next, *social avoidance* (low approach; high avoidance) is characterized by both a high desire to avoid others and a drive to be alone. Lastly, *unsociability* (low approach; low avoidance) is characterized by a heightened preference for solitude in the absence of strong avoidance motivations (or feelings of anxiety). This motivational model served as the theoretical ‘backbone’ of social withdrawal research for the last 30 years. However, as noted by Coplan and Bowker (10), research on social withdrawal focuses almost exclusively on the causes and consequences of motivations for solitude, with only a handful of studies actually measuring time alone. With this in mind, we set out to provide a review and synthesis of how solitude has been operationalized and measured in studies of children, adolescents, and emerging adults.

#### Inclusionary and exclusionary criteria

**Figure 1:**
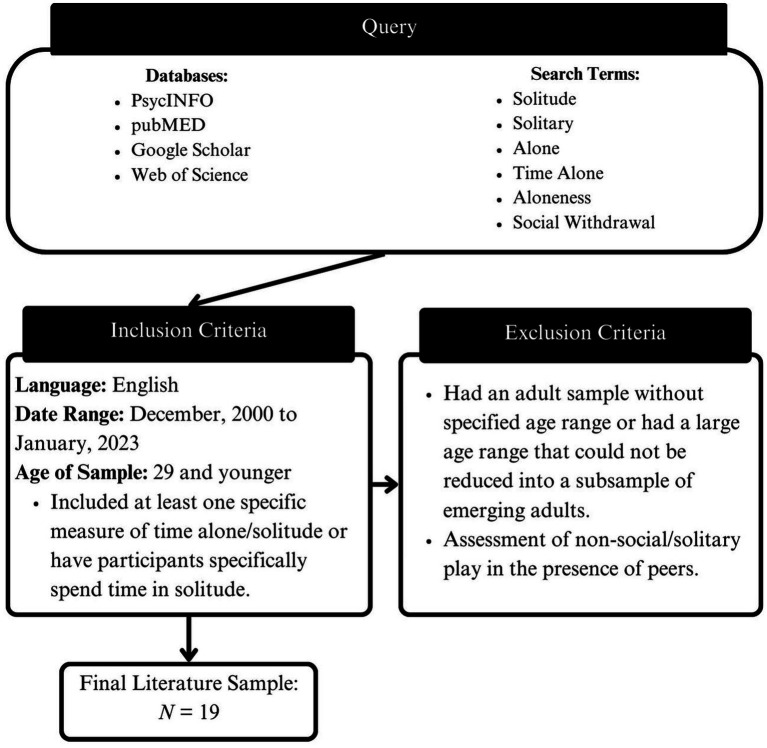
Flow diagram of the review process.

Information regarding databases and search terms used, as well as inclusionary and exclusionary criteria is presented in Figure 1. We set a temporal criterion of articles published since the year 2000. Although this excluded seminal historical research in this area [e.g., (24, 25)], we felt it was important to focus on more contemporary perspectives. Next, although we initially intended to only include studies with samples of children and adolescents, we ultimately extended this criterion to include samples of emerging adults [i.e., ages 19–29 years; (26)].

The central aim of this review was to identify original published studies (in English) measuring *solitude*. This included studies with experimental manipulations [e.g., asking participants to sit alone in an empty room, e.g., Wilson et al. (12)], as well as studies assessing solitude over a predetermined period of time [e.g., participant completion of end of day reports, e.g., (27)]. In this regard, we did not include studies including only measures of solitude motivations, such as the *Child Social Preference Scale* [e.g., “If given the choice, my child prefers to play with other children rather than alone”; (28)] or general tendencies to engage in solitary behaviors, such as the *Child Behavior Scale* [e.g., “Withdraws from peer activities”; (29)]. In this same vein, we excluded studies focusing exclusively on attitudes and beliefs about solitude using quantitative [e.g., (30)] or qualitive assessments [e.g., (31)].

Finally, there have been several previous studies in which researchers employed naturalistic observations to assess children’s non-social behaviors (e.g., reticence) and solitary play forms (solitary-passive, solitary-active) at schools/childcare centers, on playgrounds, and in laboratory playrooms (see (32), for a review). In these studies, a child is typically coded as being engaged in a ‘solitary’ activity when they are at least feet away from other children. Such behaviors, in the presence of peers, are well-established indicators of *social withdrawal* [i.e., removing oneself from opportunities for peer interaction; (3)]. However, children in this context are neither *physically* alone, nor can it be assumed that they *perceive* themselves as alone. Accordingly, for conceptual reasons, we decided to exclude such studies from our analyses.

## Analysis of studies measuring solitude

After applying exclusionary criteria, our review of the extant literature identified N = 19 empirical studies. To organize our discussion of these studies, we grouped them into three broad methodological categories: (1) experiments/manipulations (*n* = 5); (2) retrospective reports (*n* = 7); and (3) experience sampling measures (ESM; *n* = 7). Key characteristics of these studies are displayed in Table 1.

**Table 1:** Studies examining solitude from childhood to emerging adulthood.

Article		Definition	Measurement	Participants
Experiments/manipulations				
	Wilson et al. (12)	N/A	Participants sat alone in a room for 6–15 min. Across 10 studies, variations of this protocol included different settings (i.e., lab room vs. at home) and different activities (doing nothing vs. engaging in self-selected solitary activities vs. being given the option to self-administer an electric shock)	*N* = 15–146 US college students
	Buttrick et al. (33)	N/A	Participants sat alone in a room a home for 12 min, instructed to either think or engage in external activities (e.g., reading, listening to music)	*N* = 2,557 college students (Belgium, Brazil, Costa Rica, Japan, Malaysia, Portugal, Serbia, South Korea, Turkey, United Arab Emirates, and United States)
	Nguyen et al. (11)	N/A	Participants sat alone in a room for 15 min. Across 4 studies, variations of the protocol included different activities (e.g., doing nothing vs. reading) and choice (e.g., choice vs. no choice), as well as variations in thought content (e.g., positive vs. neutral)	*N* = 108–343 undergraduate students ages 18–29 years
	Hatano et al. (34)	N/A	Participants sat alone for 3–20 min. Across five studies, variations of the protocol included different settings (e.g., lab room vs. booth), time (e.g., 3 vs. 20 min), and activities (e.g., doing nothing vs. browsing Internet)	*N* = 30–63 Japanese university students (*M*_age_ = 18.92–20.02)
	Nguyen et al. (35)	N/A	Participants sat alone in a room for 5–15 min. Across three studies, variations of the protocol included different activities (e.g., doing nothing vs. sorting pencils) and instructions (e.g., autonomy-supporting vs. autonomy-controlling)	*N* = 266–369 US university students ages 18–28 years
Retrospective reports				
	Leary et al. (36)	Not operationalized	Participants indicated how many times in the last month they engaged 12 solitary activities	*N* = 204 US university students
	Coplan et al. (37)	By yourself or doing something by yourself—not including sleeping	How many times were you alone in the last week for a period lasting at least 15 min? How many total hours did you spend alone in the last week?	*N* = 379 Canadian/US university students (*M*_age_ = 19.80)
	Archbell et al. (38)	Not operationalized	Parents reported on child’s daily social activities between 6 am-8 pm (e.g., alone, with peers, with others)	*N* = 89 Canadian children ages 6–9 years
	Coplan et al. (39)	By yourself or doing something by yourself—not including sleeping	How many times were you alone in the last week for a period lasting at least 15 min? How many total hours did you spend alone in the last week?	*N* = 869 Canadian/US adolescents ages 15–19 years
	Hipson et al. (13)	By yourself or doing something by yourself—not including sleeping	How many times were you alone in the last week for a period lasting at least 15 min? How many total hours did you spend alone in the last week?	*N* = 869 Canadian/US adolescents ages 15–19 years
	Bosacki et al. (40)	Not operationalized	Participants reported how much time they spend alone during a typical day and week	*N* = 61 Canadian adolescents ages 11–18 years
	White et al. (27)	Activity not involving any direct physical or verbal interaction with others	Participants indicated whether they were mostly alone, with other people but not interacting, or with other people and interacting that day	*N* = 411 US university students ages 18–26 years
Experience sampling measures				
	Brown et al. (41)	Not operationalized	Participants indicated whether they were alone or with others 8 times a day for 7 days	*N* = 245 US university students
	Kwapil et al. (42)	Not operationalized	Participants indicated whether they were alone or with others 8 times a day for 7 days	*N* = 56 US university students (*M*_age_ = 21.2 years)
	Matias et al. (43)	N/A	Participants responded to the open-ended question “Who are you with?” Responses coded as “alone” or “not alone” (e.g., in the presence of others)	*N* = 44 Portuguese university students (*M*_age_ = 21 years)
	Wang et al. (44)	Not operationalized	Participants indicated whether they were physically alone three times a day	*N* = 28 US university students (*M*_age_ = 21.437)
	van Roekel et al. (45)	Not operationalized	Participants reported whether they were alone or with others 9 times a day for 6 days	*N* = 103 Dutch adolescents ages 13–16 years
	Thomas et al. (18)	N/A	Participants indicated if they were: (a) physically alone and not communicating with anyone; (b) physically alone and communicating with someone; (c) around people but not interacting with them; (d) around people and interacting with them; (e) around people and communicating with someone not physically present	*N* = 69 US university students ages 18–35 years
	Uziel and Schmidt-Barad (46)	Being physically alone while not actively communicating with others	Participants indicated whether they were alone or with others	*N* = 155 Israeli university students (*M*_age_ = 23.92)

Definition = operational definition of solitude provided to participants; Measurement = measurement of solitude; not all studies provided demographic/geographic information.

### Experiments/manipulations

Researchers conducting experiments on solitude aim to make causal claims regarding the implications of spending time alone. A key benefit of experiments involves the ability to randomly assign participants to different conditions (e.g., alone, with others), which increases internal study validity and provides more unbiased estimates (47). Researchers may also isolate the potentially salient aspects of solitude by controlling extraneous variables across conditions (e.g., solitary activities, timeframe, location, and autonomy). Of note, our review found that experimental studies of solitude have been conducted exclusively with emerging adults.

For instance, in a series of 11 experiments, Wilson et al. (12) investigated university students’ experiences of solitude. The first six experiments involved asking participants to sit alone in a plain room without their belongings for anywhere from 6 to 15 min, with the only instructions being to “remain in their seats and stay awake” (p. 2). Across studies, participants reported overall low levels of enjoyment, high levels of boredom, and difficulty concentrating. In one follow-up study, findings extended beyond the lab to the home setting.

In other follow-up studies, Wilson et al. (12) compared the effects of being in pure solitude (i.e., physical solitude with no distractions) to those of engaging in mundane solitary activities, such as reading or listening to music. Results indicated that participants consistently preferred engaging in solitary activities over doing nothing. Indeed, the desire to avoid doing nothing was so strong, that in one experiment, many participants (especially men) chose to self-administer a previously experienced painful electric shock instead of sitting alone with their thoughts for 15 min. Taken together, findings suggest that engaging in pure solitude is an undesirable, and even aversive, way to spend time alone.

In a subsequent study, these findings were replicated across cultures. Buttrick et al. (33) compared experiences of *thinking* versus *doing* while alone in samples of college students from 11 countries (i.e., Belgium, Brazil, Costa Rica, Japan, Malaysia, Portugal, Serbia, South Korea, Turkey, United Arab Emirates, and United States). In the thinking condition, participants were instructed to “entertain themselves with their thoughts as best as they could, with the goal of having a pleasant experience” (p. e75) and no distractions or technological devices. In the doing condition, participants engaged in external leisure activities of their choice, such as reading, watching TV, surfing the Internet, playing video-games, or listening to music. School work and routine activities were not permitted, as the activity was intended to be enjoyable. Overall and across all countries, students reported enjoying spending time alone engaged in an activity more than spending time in pure solitude. However, it should be noted that participants did not particularly enjoy either condition. Overall ratings of enjoyment averaged 4.54 and 6.35 in the thinking versus doing conditions, respectively, on a scale with a possible range from 3 to 27.

Hatano et al. (34) subsequently examined the effects of just thinking among Japanese university students. Participants in this study were assigned to sit in pure solitude in various locations (e.g., room, dark booth) without their belongings for periods ranging from three to 20 min. Before the study began, participants rated how enjoyable they *expected* the assigned activity to be. Across experiments, participants found sitting alone with their thoughts more enjoyable, engaging, and interesting, as well as less boring than they had expected. In a follow-up experiment, participants were assigned to either spend 20 min in pure solitude or browsing Internet news sites alone. Although participants predicted they would enjoy the browsing activity more than the waiting activity, results showed that experiences did not differ between the conditions. So, there is at least some evidence to suggest that emerging adults enjoy being alone with their thoughts more than they think! As well, doing at least some specific activities while alone (i.e., browsing Internet news sites) is not necessarily better than doing nothing.

Having said that, *pure* solitude represents the most restrictive operational definition of solitude, eliminating other factors that may impact upon experiences while alone (e.g., location, choice of activity). However, as a result, this approach externally imposes the conditions of solitude (i.e., where, how long, doing what) and notably confounds context (solitude) with tolerance of inactivity. Given these constraints, it is perhaps not surprising that young people experience pure solitude so negatively. Another factor to consider is how participants’ experiences of solitude are quantified. For example, in the aforementioned studies (33, 12), enjoyment of solitude was assessed by averaging participants’ reports of how enjoyable, entertaining, and boring (reverse scored) the activity was. However, in terms of individuals’ affective experiences during solitude, emerging evidence suggests that it is important to consider different combinations of *valence* (i.e., positive vs. negative) and *arousal* (i.e., activation vs. deactivation) (11, 35).

For example, in a series of studies, Nguyen et al. (11) instructed emerging adults to sit alone for 15 min without engaging in other activities. The researchers then compared the effects of this pure form of solitude to those of engaging in external solitary activities (e.g., reading). Across experiments, results supported a *deactivation* effect of solitude, such that spending time in solitude (regardless of whether participants engaged in an external activity) led to decreased high arousal positive affect (e.g., happiness) and increased low arousal negative affect (e.g., loneliness), along with increased low arousal positive affect (e.g., relaxation) and decreased high arousal negative affect (e.g., anger).

In a third experiment, the researchers randomly assigned participants to conditions differing based on both choice and thought content. In the choice condition, participants were instructed to “think during their time alone, but that they could choose to think either positive or neutral thoughts” (p. 96). In the no choice condition, participants were assigned to think either positive or neutral thoughts. Lastly, the control condition mirrored the earlier pure solitude condition. Although results again indicated that solitude had a deactivating effect, thinking positive thoughts (in either of the choice groups) inhibited the reduction in high arousal positive affect. These findings suggest that despite not being enjoyable, pure solitude confers some benefits (particularly in terms of increased restoration) and that (at least some of) the risks associated with solitude can be mitigated through regulating one’s thoughts.

In a final experiment, Nguyen et al. (11) collected daily diary data over 2 weeks with using a *switching-replication* design to examine the implications of daily solitude on emerging adults’ affect and well-being. The researchers randomly assigned participants to either “spend 15 min in solitude (i.e., without electronic devices or activities) sometime during each day of the first week of the study” (p. 100), or not engage in solitude during that week. During the second week of the study, the two groups switched tasks. At the end of each day, participants completed measures of affect, vitality, satisfaction, and stress. Consistent with the notion that emerging adults do not think they will enjoy spending time alone, almost a quarter of participants reported being non-compliant during supposed episodes of solitude (e.g., mentioning sleeping, eating, doing schoolwork, interacting with others remotely or in person, or engaging with technology). Notwithstanding, results again indicated a deactivating effect of solitude for high-arousal positive affect and high-arousal negative affect. Solitude also predicted lower vitality, which is an energizing state. Interestingly, there was a *spillover effect* of solitude on arousal, such that participants who engaged in solitude during the first week of the study remained more deactivated during the second week. Although solitude was not associated with low arousal affective outcomes overall, participants with low autonomy for solitude reported lower low-arousal positive affect and higher low-arousal negative affect, as well as increased stress and reduced satisfaction after engaging in solitude. Participants with high solitude autonomy, on the other hand, reported higher low-arousal positive affect and less stress after engaging in solitude. Findings suggest that spending time alone is not only less harmful, but also more beneficial, when young people feel motivated to choose solitude for positive reasons.

As aforementioned, experimental studies typically impose conditions on participants’ experiences of solitude. However, when aspects of solitude are externally constrained, they are more likely to result in negative experiences [e.g., (11)]. In this regard, Nguyen et al. (35) recently investigated whether the affective implications of solitude could be improved by enhancing autonomous motivation for solitude among emerging adults. In two studies, participants were first instructed to sit in a room without their belongings for 15 min. During this phase, the researchers manipulated participants’ autonomy for solitude through use of either autonomy-supportive or autonomy-controlling language. Autonomy-controlling instructions included language such as “you must” or “you should,” and stressed that the experimenter “expected” the participant to sit alone without engaging in other activities (p. 3). Autonomy-supportive instructions included language such as “I invite you to” and “you can,” and the researchers emphasized that “different people might have different reactions to the activity so that participants could feel free to explore their feelings with a sense of choice” (p. 3). Finally, participants were presented with a *free choice* period, wherein they chose between sitting alone with their thoughts and sorting pencils for 10 min.

Consistent with Nguyen et al.’s (11) findings, results indicated that high arousal positive and negative affect decreased after participants engaged in pure solitude in both studies. However, although low arousal positive affect also increased in both studies, low arousal negative affect was found to increase in the second study, but not the first. During the free choice period, participants were much more likely to sort pencils than sit with their thoughts. These findings further support the idea that although engaging in pure solitude may offer benefits in terms of emotion regulation (11), doing nothing is not appealing to emerging adults (33, 12). When given the choice, even mundane (e.g., pencil sorting) and aversive (e.g., self-administration of an electric shock) activities are preferred. Interestingly, although the manipulation of autonomy for solitude was successful, autonomous motivations for solitude did *not* play a significant role in participants’ responses to solitude.

Taken together, studies relying on experimental methods highlight a key theme in solitude research: young people clearly prefer doing something over doing nothing while alone (although engaging in pure solitude may confer affective benefits related to increased peace and relaxation). As such, it is important to consider solitary activities when understanding solitary experiences. These findings also provide some insight into the importance of choice. Having high autonomy related to solitude may not only protect against the potential negative outcomes of time alone, but it may confer unique affective benefits. Still, evidence regarding the importance of choice is mixed, with one study showing that outcomes of solitude remained consistent regardless of differences in autonomous motivations for solitude (35).

Although experimental studies allow for a high degree of precision and control (which is important for isolating the effects of solitude), such studies may lack external validity (48). Indeed, given what is known regarding young people’s perceptions of pure solitude, it is unlikely that adolescents and emerging adults spend considerable time alone with their thoughts in real life. In this regard, solitude may look (and function) quite different outside of the laboratory setting. Moreover, when aspects of solitude are externally constrained, they are more likely to result in negative experiences (11). As such, it is also important to examine *naturally occurring* solitude.

### Retrospective reports

To explore solitude in naturalistic settings, some researchers have examined *retrospective* reports of time spent alone. Our review revealed studies asking participants to recall instances of solitude over specified periods of time ranging from the end of the day to the previous week. Whereas experimental studies of solitude focused exclusively on emerging adults, retrospective studies also include samples of adolescents and children. These studies differ from the previously described experimental designs insofar as they assess naturally occurring episodes of solitude. In this regard, the results can speak more generally to the association between time spent alone and adjustment.

For example, Coplan and colleagues (13, 37, 39) assessed retrospective reports of both episodes of solitudes (i.e., how many times were you alone in the last week for a period lasting at least 15 min?) and time spent alone (i.e., how many total hours did you spend alone in the last week?) in samples of adolescents and emerging adults. Time alone was operationalized for participants as “by yourself, or doing something by yourself, not including sleeping” (e.g., (37), p. 20). An aggregate score of solitude was computed by averaging these two items.

Using this measure, Coplan et al. (37) found that weekly solitude was positively related to emerging adults’ preference for solitude and loneliness (but not stress), and negatively related to feelings of aloneliness (i.e., negative feelings that arise from the perception that you are not spending enough time alone). Interestingly, aloneliness was highest among emerging adults who reported a higher preference for solitude yet spent little time alone. In a second sample, time alone was also positively related to emerging adults’ depressive symptoms and stress. However, among emerging adults who reported feeling more alonely, the link between time alone and depressive symptoms was attenuated. These findings suggest that when young people are dissatisfied with the amount of time they have been spending alone, seeking solitude need not be risky. Moreover, finding time away from others may be particularly important for those with high preference for solitude.

In a later study using this measure with adolescents, Coplan et al. (39) reported that, overall, self-reported time alone was negatively related to sociability and positive affect, and positively related to shyness and negative affect. Still, results from follow up person-oriented analyses further emphasized that not all time alone is created equal. Four sub-groups of adolescents were identified that spent comparatively more time alone than their peers. For two of these groups, frequent solitude was associated with maladaptive motivations and negative emotional experiences. Specifically, the group labeled *shy*-*withdrawn* was characterized by high shyness and high sociability, as well as high negative affect, whereas the *socially avoidant* group reported high shyness and low sociability, as well as high negative affect and low positive affect. In contrast, two other groups reported higher time alone, but appeared more normative and positively adjusted. Specifically, the *unsociable* group reported low sociability, but also low negative affect, whereas the group labeled *balanced* was characterized by the unique combination of high sociability, low shyness, and high positive affect. Of note, intrinsically motivated solitary activities were reported as more common among *unsociable* and *balanced* adolescents, which the authors postulated may have accounted for lower reported aloneliness among these groups.

Finally, in another study of adolescents using the same measure, Hipson et al. (13) reported that time alone was positively related to preference for solitude and negative affect, as well as negatively related to positive affect. It should be noted, however, that the link between time alone and positive affect was *curvilinear*. That is, at less than 1 h per day, time alone was not correlated with positive affect. At moderate levels, then, perhaps time away from others is less harmful for young people [see also (6)].

Hipson et al. (13) provided further evidence that not all time alone is the same. Participants were asked to list the three things they did the most when they were alone over the last week. The most commonly endorsed solitary activities included passive screen time (e.g., Netflix; 41%), homework (40%), and listening to music (23%). Although daydreaming was reported by 18% of adolescents, other types of thinking activities (e.g., negative thinking, planning) were more uncommon (~5%). Moreover, few participants reported engaging in meditation (4%), relaxing (4%), or doing nothing (6%), which provides further support for the notion that pure solitude is not favorable (12, 33, 35).

Results from subsequent person-oriented analyses revealed three sub-groups of adolescents characterized by their engagement in different patterns of solitary activities. The largest group included over half the sample (53%) and was comprised of adolescents who typically engaged passively with technology (e.g., watching TV) or did homework while alone. Adolescents in the second-largest group (31.7%) tended to spend their solitary time engaged in more active forms of technology use (e.g., social media and video games), as well as hobbies, homework, and listening to music. Lastly, the smallest group (15%) included adolescents who spent time alone primarily engaged with their thoughts.

When comparing solitary activity groups on indices of well-being, findings revealed that adolescents who spent considerable time in pure solitude (e.g., thinking, ruminating) experienced increased depression, anxiety, and loneliness as compared to those who engaged in other solitary activities. Indices of adjustment did not differ between adolescents who spent time alone passively engaged with technology and those who participated in more active activities, suggesting that doing something (regardless of what that something is) is better than doing nothing. Notably, the groups did not differ in preference for solitude.

Bosacki et al. (40) employed a similar methodological approach with a sample of adolescents during the COVID-19 pandemic, but included questions regarding experiences over the course of a *typical* week (i.e., how many times are you alone during a typical week?) and day (i.e., how many times are you alone during a typical day?). Participants also indicated whether they were typically physically alone more than with others and whether it was their choice to be alone (i.e., yes, no). However, ‘alone’ was not operationally defined for participants. Results revealed that adolescents engaged in one or two episodes of solitude lasting at least 15 min each day and spent approximately 8 h alone each week. Older adolescents also reported spending more time alone than younger adolescents.

Almost 70% of adolescents indicated that they were with others more often than alone and 65% reported spending time alone by choice. These findings indicate that, more often than not, adolescents seek solitude volitionally. Bosacki et al. (40) also reported that weekly (but not daily) solitude was positively related to preference for solitude, suggesting that adolescents with higher preference for solitude may spend more time away from others. Engaging in solitude for external reasons (but not by choice) was associated with higher social anxiety and negative affect, as well as poorer self-perceptions. Thus, agency may be critical in determining outcomes of time alone.

White et al. (27) asked undergraduate students to report on daily time alone over a 7-day period. At the end of each day, participants indicated whether they were mostly alone, with other people but not interacting with them, or with others and interacting for five timeframes (i.e., waking up to 9:00 am; 9:00 am to 12:00 pm; 12:00 to 3:00 pm; 3:00 to 6:00 pm; 6:00 to 9:00 pm). Among the results, emerging adults who spent more time alone overall experienced increased high arousal positive affect *when with others*. Moreover, spending more time alone than usual was associated with increased low and high arousal positive affect when with others on the same day at the within-person level. Interestingly, on days when participants spent increased time alone, shyness and avoidance were both associated with higher anxious affect (and avoidance with higher low arousal negative affect) during social encounters, whereas unsociability was associated with lower anxious and low arousal negative affect during social encounters. Taken together, although time spent alone may be beneficial for most emerging adults, those high in shyness or avoidance may struggle to re-integrate into social settings after periods of extended solitude.

White et al.’s (27) findings provide some of the first empirical evidence to support a widely held theoretical perspective that time away from others provides space for renewal, particularly for those who enjoy solitude. Related to this notion, Leary et al. (36) examined solitary activities in a sample of undergraduate students. Participants indicated how many times in the last month they engaged in a list of 12 activities “by themselves” (p. 62–63). Frequency and enjoyment of solitary activities was predicted more by increased solitropism (i.e., desire for aloneness) than sociotropism (i.e., desire to avoid others). The authors speculated that by spending time alone, individuals with higher preference for solitude may free themselves from social expectations and manage their arousal and stress levels. In a way, then, solitary activities may act as a social battery charger, bringing more balance to young people’s lives and better enabling them to thrive interpersonally.

Finally, our review of the literature revealed only a single study where researchers measured time alone in a sample of children. Archbell et al. (38) conducted a series of end of day telephone interviews with parents of early elementary school students (grades 1–3). Interviews were conducted on three different weekdays and two weekend days over 4 months. For each interview, parents reported the social context of their child’s daily activities in 2-h intervals between 6 am and 8 pm (e.g., alone, with peers, with others). Results revealed that, on average, children spent only about 10% of their time outside of school alone. Parents also reported that children in Grade 3 spent significantly more time alone than children in Grade 1. Associations between time alone and well-being indices were not examined.

Results from retrospective studies further highlight the importance of considering differences in autonomy and activity when examining solitude. Engaging in solitude by one’s own volition may protect against the negative effects of increased time alone in adolescence (40). Further, findings from these studies highlight that individuals may choose to be alone for various reasons. Adolescents and emerging adults who are motivated to approach solitude for positive reasons (e.g., enjoyment), may benefit from taking time alone to recharge, whereas those seeking solitude to avoid social situations perceived as anxiety-provoking or unpleasant may be particularly at risk for negative outcomes (27, 37, 39).

Finally, findings from retrospective studies suggest that, similar to their emerging adult counterparts, adolescents do not favor pure solitude. Rather, they prefer spending time alone engaged with technology, homework, or hobbies (13). When it comes to the implications of solitude, doing something (particularly when that something is intrinsically motivated) is better than doing nothing (13, 39). It should be noted that only one retrospective study considered potentially important differences in valence and arousal when examining the affective outcomes of solitude.

### Experience sampling measures

One limitation to retrospective approaches is that individuals may struggle to accurately recall how much time they spent alone (or what they did) over a period of days to weeks (49). To combat recall issues and enhance ecological validity, researchers have begun using ESM to examine naturally occurring experiences of solitude *as they unfold in real time*. Such studies (which often rely on smartphones or other technological devices) have become especially popular with the rise of technology (50). ESM studies provide multiple observations per person and allow researchers to test hypotheses at within- and between-person levels. As such, this approach to conducting research allows for a rich understanding of young people’s social experiences (51).

Our review revealed only one study using ESM methods to measure solitude among adolescents. Van Roekel et al. (45) assessed adolescents’ (aged 13–16 years) feelings of loneliness across social contexts and locations. Participants responded to nine random beeps a day for 6 days. After each notification, adolescents indicated whether they were alone or with others. ‘Alone’ was not operationalized. Those indicating that they were in company also responded to an open-ended question regarding who they were with. The researchers then categorized responses to family (e.g., parents or siblings), friends, classmates, or others (e.g., team-mates or teachers).

Consistent with previous retrospective studies, adolescents were in company more often than they were alone (40, 46). Moreover, momentary solitude predicted higher levels of loneliness across genders and locations. When comparing the effect of solitude across two consecutive assessments, results revealed that being alone at the previous assessment had a prolonged negative effect on adolescents’ loneliness when they were with family at the next assessment. However, when adolescents were with friends at the next assessment, they reported feeling less lonely. Van Roekel et al. (45) suggest that this *relief effect* may stem from adolescents’ desire to be around friends. Results here may also provide some support for White et al.’s (27) recent assertion that increased solitude helps emerging adults recharge, thereby allowing them to experience more enjoyment when interacting with others the same day. However, motivations for solitude and solitary activities were not considered.

In terms of studies with emerging adults, Kwapil et al. (42) examined links between social anhedonia and experiences of solitude in a small sample of female university students using ESM. Participants received alerts using palm pilots eight times a day over 12 h (12:00 pm to 12:00 am) for 7 consecutive days. After each notification, participants had up to 5 min to begin the assessment, where they indicated whether they were alone or with others. ‘Alone’ was not operationally defined. Participants then responded to questions regarding their experience of the social context, as well as positive and negative affect. Results revealed that social anhedonia was associated with a greater likelihood of being alone at the time of assessment, but also with choosing to be alone and enjoying solitude. Interestingly, although solitude was linked to higher negative affect (but not positive affect) overall, participants higher in social anhedonia reported lower negative affect and higher positive affect while alone. Findings are consistent with the idea that social motivations can moderate the impact of solitude on affective well-being.

Brown et al. (41) also examined social anhedonia in a university sample using ESM. Participants were notified eight times via palm pilot (between noon and midnight) for 7 days. After each notification, participants had 5 min to begin completing the questionnaire, which assessed affect (i.e., positive and negative affect, anxiety, sadness, and self-consciousness), social contact (i.e., alone vs. with others), cognitions, and activities. ‘Alone’ was not operationalized. Among the results, being alone was associated with higher momentary negative and lower positive affect compared to being with others. Social anhedonia predicted increased time alone and increased desire to be (and stay) alone, as well as disengagement in social contexts. Social anxiety, on the other hand, predicted increased desire to be alone when with others (especially acquaintances). However, social anxiety was not related to time alone or lower desire to be with others when in solitude. In addition, social anxiety was related to feelings of social rejection, but social anhedonia was not. Consistent with Kwapil et al. (42), these findings suggest that young people high in social anhedonia prefer solitude. Findings regarding social anxiety tell a different story, wherein socially anxious individuals want to engage with others; however, their desire and level of comfort in doing so is related to relationship closeness.

Matias et al. (43) examined how momentary solitude relates to affective experiences and cortisol in a sample of female college students. Participants received random notifications through electronic pagers eight times a day (between 8:00 am and 11:00 pm) for six consecutive days. In each instance, participants responded to the open-ended question, “Who are you with?” Responses were coded as either “alone” (e.g., alone, alone in a room) or “not alone” (e.g., alone in a crowd, with friends, with colleagues). Participants also provided momentary ratings of their positive (i.e., happy, joyful, cheerful, in a good mood) and negative (i.e., sad, bored, lonely) affect, as well as anxiety. Compared to being with others, being alone was linked to lower momentary positive affect and greater negative affect (but was unrelated to anxiety). The researchers also found that being in solitude directly predicted higher cortisol levels compared to being with others, especially among participants high in general negative affect or low in general positive affect.

Uziel and Schmidt-Barad (46) used ESM to specifically examine how *choice* impacts emerging adults’ experiences of being alone versus with others. Participants were notified three times a day via text (i.e., morning, noon, and evening), 5 days a week over a two-week period. After each notification, participants indicated whether they were alone (i.e., physically alone and not actively communicating with others) or with others (i.e., in the same physical space and/or actively communicating with others). Consistent with adolescents’ retrospective reports (40), emerging adults in this study spent more time with others than alone (63% vs. 37%) and indicated that most of the time (73%), they were alone by choice. Among other results, being in solitude (compared to being with others) and being in non-chosen settings (compared to being in chosen settings) were linked to lower positive affect, satisfaction with life, and meaning, as well as higher negative affect overall. Social context also moderated the effect of choice, such that emerging adults reported poorer well-being when in solitude, regardless of whether they chose to be there or not. In contrast, when participants were with others, having a choice was beneficial for well-being. On the surface, these findings suggest that solitude is detrimental regardless of autonomy in decision-making. However, the researchers did not account for social motivations. Individuals may choose to spend time away from others for a variety of reasons (e.g., strong avoidance tendencies, enjoyment of solitude, social fears). As has been revealed in experiments and retrospective reports, differences in feelings about solitude may play an important role in the experience of being alone.

As aforementioned, it is now possible (and commonplace) to be physically alone while virtually engaging with others. Retrospective studies of solitude suggest that many young people spend time alone interacting with technology (13). Such findings are further supported through ESM studies. For example, Wang et al. (44) used ESM to examine the role of solitude in emerging adults’ needs and media use. Participants were notified three times a day (i.e., lunchtime, early evening, before bed) via cell phone or another device. After each notification, participants reported whether they were physically alone. ‘Alone’ was not operationalized for participants. They also reported on their social media (e.g., Facebook, Twitter, YouTube, email) and other media (e.g., television, radio, magazines) use over the past several hours. Among the results, solitude was associated with increased social and especially other media use.

Thomas et al. (18) examined how momentary solitude relates to mood regulation abilities and identity development in university students. The researchers differentiated between being in true solitude (i.e., physical solitude without digital communication or social media) and physical solitude while engaged with others virtually. After downloading an app on their smartphone, participants were randomly notified seven times a day (during a 16-h timeframe) for 7 days. In this study, the authors did not simply differentiate between “alone” and “with others.” Rather, participants selected one of five options that best fit their social status when prompted: (1) physically alone and not communicating with anyone (truly alone), (2) physically alone but also communicating with someone (on device alone), (3) around people but not interacting with them (around others), (4) around people and interacting with them (social), or (5) around people and also communicating with someone who was not physically present (social while on device). Participants who reported communicating with others when prompted also identified whether the means of communication was face-to-face, phone, letter, video, text, instant message, or social media platform.

Among the results, participants reported being truly alone 19% of the time, alone in the presence of others 17% of the time, and on their devices alone 9% of the time. Although the most common form of communication was face-to-face (40%), participants also frequently communicated via text (13%). Moreover, participants were already on their phone about a quarter of the time (26%) they were notified to complete momentary assessments (and on social media sites or messaging others 13% of the time). These results suggest that young people spend considerable time engaging with others virtually. However, neither time spent truly alone nor time spent alone on a device were related to indices of well-being or motivations for solitude overall.

A follow-up cluster analysis showed that introverts with higher preference for solitude demonstrated positive psychosocial adjustment (i.e., high identity development, autonomy, and positive relationships, and low loneliness) and low negative motivation for solitude. Interestingly, they also spent the most time in true solitude. Introverts without high preference for solitude, on the other hand, experienced more negative motivations for solitude, spent more time on social media, and demonstrated poor psychosocial adjustment (i.e., low identity development and high loneliness). In addition, being alone on one’s device was associated with improved momentary affect when compared to being in true solitude; however, only among participants that did not want to be alone. These results collectively provide further support for the importance of considering individuals’ internal motivations in conjunction with solitary activities when examining experiences of solitude. Spending increased time alone need not hinder psychosocial adjustment and well-being, if one is happy to be there.

Findings from ESM studies are largely consistent with conclusions emerging from our review of experimental and retrospective studies and provide additional support for the importance of considering the roles of autonomy, motivations, and solitary activities in the correlates of solitude.

## Discussion

### Measuring solitude: what matters?

In this review, we reviewed empirical studies including measures of solitude among children, adolescents, and emerging adults since the year 2000. These studies included three main methodological approaches: (1) experiments/manipulations; (2) retrospective reports; and (3) ESM. Each approach affords unique advantages and disadvantages, and thus, continued use of these methodologies is warranted.

Regardless of what methods are employed, however, more studies on solitude are needed. In over 20 years, we uncovered only 19 empirical studies either instructing participants to engage in solitude or measuring naturally occurring instances of solitude. Moreover, most of these studies were conducted with samples of emerging adults, only a handful included samples of adolescents, and, astonishingly, after excluding observations of non-social play in the presence of peers, we found only a single study assessing solitude in children.

There may be methodological reasons for the lack of studies in childhood. For example, in terms of experimental designs, placing children (particularly young children) in a room alone may evoke safety concerns (52) and raise other ethical issues. Related to retrospective measures, research on temporal memory suggests that young children may not accurately recall how much time they spend alone in a day (53). As well, conducting ESM research with children evokes unique challenges, most notably non-compliance rates of over 50% (54).

There also remains considerable variation *within* measurement approaches employed in existing studies, and it is unclear how such variations might impact research results. For example, across all study types, researchers provide varying (or often no) operational definitions of ‘solitude’ for participants. Participants may have different conceptualizations and definitions of what it means to be alone (31), which may impact upon study findings. In *experimental* studies of solitude, it may be important to consider how long participants are instructed to spend alone [e.g., times ranged from 6 to 20 min; (12, 34)], and what participants can do during that time [e.g., nothing, read, or an activity of choice; (12), 33]. In *retrospective* studies, researchers have asked participants to report not only how much time they spent alone over varying specific time periods [e.g., last day vs. last week; (27, 39)], but also in a ‘typical’ day/week (40). In ESM studies, researchers typically ask participants to indicate whether they are alone or with others at random times over the course of a day (43, 46). Factors including number of daily assessments, types of items (e.g., single-item scales vs. multi-item scales), day of the week (i.e., weekday vs. weekend), and lag time between signal and response may all play a role (55).

Differences in outcomes assessed may also be of consideration. Studies typically consider positive and negative affect without distinguishing between valence and arousal. Emerging evidence indicates that solitude has a deactivating effect, wherein high arousal emotions are reduced and low arousal emotions are enhanced (11, 35). As such, examining links between solitude and positive and negative affect without considering arousal may paint an inaccurate picture of the outcomes of seeking time away from others. In adolescence, solitude also provides a context to work through important developmental tasks, such as gaining autonomy and forming strong identities (13). Researchers could thus expand beyond affective outcomes to include factors related to autonomy and identity formation. Other important outcomes to include may be academic and socio-emotional skills.

Despite these issues, overall and across methodologies, time alone was associated with negative outcomes for young people. However, even after considering measurement issues, experiences and implications of solitude vary according to several other factors. We discuss these briefly in the final section of this review, with an additional eye towards future research.

### Measuring solitude moving forward: what else matters?

#### Doing nothing versus doing something(s)

When it comes to spending time alone, it is clear that engaging in pure solitude is not a sought out or particularly enjoyable experience for young people (33). Instead, adolescents and emerging adults generally prefer to spend time alone engaged in external activities, such as leisure activities, homework, and both passive and active technology use (13). In general, doing something while alone is more adaptive than doing nothing (13). Even adolescents who primarily engage in passive technology use (e.g., Netflix) while alone appear to be functioning quite well (13). Using social media while alone has also been linked to higher momentary well-being among emerging adults who would prefer to be with others (18).

Despite not being enjoyable, there are some benefits to engaging in pure solitude, particularly regarding emotion regulation (35). Of note, the content of one’s solitary thoughts might be an important factor to consider. Results from several studies suggest that pure solitude is experienced negatively regardless of whether one is engaged in positive (e.g., daydreaming, planning) or negative thinking [e.g., ruminating; (13, 33)]. However, Nguyen et al. (11) found that thinking positive (but not neutral) thoughts inhibited the deactivation of high arousal positive affect. Taken together, solitary activities are heterogeneous and distinctive (13, 14), and *what you do* when you are alone matters in terms of experiences and implications of solitude.

#### Autonomy and motivations

It has been posited that choosing to spend time alone is beneficial in terms of enhancing creativity, self-reflection, and identity development (6). Our review suggests that in choosing when and how to engage in solitude, youth may also exercise their autonomy (13). Although adolescents and emerging adults typically engage in solitude volitionally (46), the impact of choice on young people’s solitary experiences was mixed. Some studies suggest that choosing to be alone (as opposed to being alone for external reasons) protects against the negative outcomes of solitude (18), whereas others indicate that solitude hinders positive development regardless of autonomy in decision-making (46). Still, individuals choose to spend time in solitude for different reasons.

Previous research has focused predominantly on the implications of different motivations for seeking solitude, including shyness, unsociability, and social avoidance (10). Our review also uncovered some evidence to suggest that differences in motivations for solitude moderate the experience and impact of being alone. For example, for young people with higher unsociability (i.e., non-fearful preference for solitude), seeking high quality time away from others may be restorative (27) and lead to positive outcomes (18). In contrast, seeking solitude as an escape from unpleasant or anxiety-provoking social contexts (e.g., shyness, social avoidance) may inhibit the benefits that come with choosing to be alone and make it more difficult to re-integrate socially later on (18, 27).

#### Gender differences

Some evidence suggests that adolescent boys spend more time alone than girls (13, 45), but results from a meta-analysis indicate no gender differences in loneliness across the lifespan (56). Notwithstanding, the *implications* of choosing to spend time alone may be worse for boys because solitary activities violate stereotypical gender norms regarding male dominance and social assertion (57). There is support of this notion, with results from several studies indicating that socially withdrawn boys evoke more negative responses from peers [e.g., (58)]. However, other results are mixed or even indicate more negative effects for socially withdrawn girls [e.g., (59)]. Further research is required to elucidate gender differences in other aspects of solitude, including when time alone might be differentially beneficial (or problematic) for boys versus girls.

#### Development beyond emerging adulthood

This review synthesized solitude research from childhood throughout emerging adulthood. Apart from our ‘call to arms’ for more of this research in children and adolescents, we would also like to highlight the continuing need for research beyond the emerging adult years. For example, *established* adulthood (i.e., age 30–45 years) represents a developmental stage characterized by a greater focus on career building and expanding one’s family (60). Of note, established adults report greater preference for solitude than emerging adults (61) but may spend less time alone (62). This is worth further exploration, as aloneliness is associated with increased stress, negative affect, and symptoms of depression (37).

Beyond established adulthood, there has been a strong focus on social isolation and loneliness (as well as aspects of solitude) among the elderly [e.g., (63, 64)]. Findings from these studies highlight some of the themes that we have discussed. For example, Tse et al. (62) found that unchosen solitary experiences were associated with lower quality momentary experiences among older adults, whereas chosen solitary activities were positively associated with indices of well-being or quality of life.

Other studies offer more novel insights. For example, Lay et al. (65) identified individual characteristics beyond social motivations contributing to variation in older adults’ experiences of solitude (e.g., social self-efficacy, rumination). Luo et al. (66) also found initial evidence to suggest that alternating between episodes of solitude and socializing promotes higher life satisfaction among older adults. It remains to be seen how these ideas might be applied to research with children and adolescents.

#### Measuring solitude in context

Finally, it will be critically important to consider solitude within broader societal and cultural *contexts*. The COVID-19 global pandemic resulted in lockdowns and social distancing across the world. We are only beginning to understand the profound impact of these experiences on young people’s mental health and well-being (8). Most studies focus on feelings of loneliness and social isolation (67, 68), but several have specifically explored experiences of solitude (69, 70). This preliminary work raises many interesting possibilities for future research. For example, did individuals who enjoy solitude fare better during times of imposed social isolation (71)?

In addition, thanks to advancements in contemporary technology, young people can now be (and often are) physically alone but virtually engaging with others (13). As aforementioned, having a sense of autonomy can enhance the benefits of solitude, whereas spending time alone for externally imposed reasons is more likely to lead to negative outcomes (11). This leads to the question, what are the implications of engaging in *involuntary* digital solitude? Real life experiences of exclusion have been found to lead to solitude. For example, Ren and colleagues (72), Ren et al. (73) have demonstrated that experiences of ostracism lead to increased preference for solitude and solitude-seeking behaviors. Similarly, Beeri and Lev-Wiesel (74) found that real-life experiences of social rejection were related to increased psychological distress and social avoidance in adolescents. It has yet to be determined if *digital* rejection also leads to more negative solitary experiences both on- and off-line.

In turns of broader contexts, our review uncovered studies measuring solitude across a range of cultures. Notwithstanding, future research should continue exploring similarities and differences in solitude globally. For example, whereas preferring to do something over nothing while alone may be culturally universal (33), there are differences across countries in the correlates of motivations for solitude (75). There is still much to learn about how cultural norms regarding group orientation, privacy, encouragement of independence, and other relevant factors influence experiences of solitude (76).

Over the last two decades, a growing number of empirical research studies have explored the psychology of solitude in children and youth. However, a lack of consensus regarding conceptualizations, operational definitions, and measures of solitude continues to pose significant challenges. Moreover, there is a pressing need for studies exploring the characteristics and implications of children’s time spent alone outside of school. Such studies will help to clarify for whom, when, how, and under what circumstances, solitude might confer costs versus benefits for child and adolescent development and well-being.

## Author contributions

AM, TC, and LP conducted the literature searching. AM analyzed the studies. AM and RC wrote the manuscript with help from TC and LP on future directions. AM, RC, TC, and LP edited the manuscript.

## Funding

This research was funded by SSHRC Insight Grant #435-2017-0849.

## Conflict of interest

The authors declare that the research was conducted in the absence of any commercial or financial relationships that could be construed as a potential conflict of interest.

## Publisher’s note

All claims expressed in this article are solely those of the authors and do not necessarily represent those of their affiliated organizations, or those of the publisher, the editors and the reviewers. Any product that may be evaluated in this article, or claim that may be made by its manufacturer, is not guaranteed or endorsed by the publisher.
